# Mutations in desmoglein 1 cause diverse inherited palmoplantar keratoderma phenotypes: implications for genetic screening

**DOI:** 10.1111/bjd.14973

**Published:** 2017-04-02

**Authors:** M.‐L. Lovgren, M.A. McAleer, A.D. Irvine, N.J. Wilson, S. Tavadia, M.E. Schwartz, C. Cole, A. Sandilands, F.J.D. Smith, M. Zamiri

**Affiliations:** ^1^ Department of DermatologyUniversity Hospital CrosshouseKilmarnockU.K; ^2^ Department of DermatologyOur Lady'sChildren's Hospital CrumlinDublinIreland; ^3^ National Children's Research CentreChildren's Hospital CrumlinDublinIreland; ^4^ Clinical MedicineTrinity College DublinDublinIreland; ^5^ Dermatology and Genetic MedicineDivision of Biological Chemistry and Drug DiscoveryUniversity of DundeeDundeeU.K; ^6^ Pachyonychia Congenita ProjectSalt Lake CityUTU.S.A; ^7^ Division of Computational BiologySchool of Life SciencesUniversity of DundeeDundeeU.K; ^8^ Alan Lyell Centre for DermatologyQueen Elizabeth University HospitalGlasgowU.K

## Abstract

The inherited palmoplantar keratodermas (PPKs) are a heterogeneous group of genodermatoses, characterized by thickening of the epidermis of the palms and soles. No classification system satisfactorily unites clinical presentation, pathology and molecular pathogenesis. There are four patterns of hyperkeratosis – striate, focal, diffuse and punctate. Mutations in the desmoglein 1 gene (*DSG1*), a transmembrane glycoprotein, have been reported primarily in striate, but also in focal and diffuse PPKs. We report seven unrelated pedigrees with dominantly inherited PPK owing to mutations in the *DSG1* gene, with marked phenotypic variation. Genomic DNA from each family was isolated, and individual exons amplified by polymerase chain reaction. Sanger sequencing was employed to identify mutations. Mutation analysis identified novel mutations in five families (p.Tyr126Hisfs*2, p.Ser521Tyrfs*2, p.Trp3*, p.Asp591Phefs*9 and p.Met249Ilefs*6) with striate palmar involvement and varying focal or diffuse plantar disease, and the recurrent mutation c.76C>T, p.Arg26*, in two families with variable PPK patterns. We report one recurrent and five novel *DSG1* mutations, causing varying patterns of PPK, highlighting the clinical heterogeneity arising from mutations in this gene.

## Abstract 2

**What's already known about this topic?**

- Twenty‐three mutations in the desmoglein 1 gene (*DSG1*) have been described in 25 families with palmoplantar keratoderma (PPK).
- The majority have striate palmar involvement with focal plantar keratoderma, but isolated focal and diffuse PPKs have been reported.

**What does this study add?**

- *DSG1* mutations can present with variable phenotype within the same family.
- Environmental factors, such as manual labour, may alter the clinical appearance of PPK.
- A lower threshold should be considered for *DSG1* screening in nonstriate PPK where an underlying keratin mutation has not been identified.

The inherited palmoplantar keratodermas (PPKs) are clinically and genetically heterogeneous genodermatoses, characterized by epidermal thickening of the palms and soles. Many keratodermas are restricted to these sites, but other cutaneous and extracutaneous features can occur.^1^ Traditionally, classification is based on the pattern of hyperkeratosis, notably diffuse, focal, striate and punctate. Identification of the genetic basis of these disorders has resulted in a molecular‐based classification;^2^ however, no single classification system satisfactorily unites the clinical presentation, pathology and molecular pathogenesis.

Striate PPK (SPPK) is a rare, mainly autosomal dominant disorder characterized by linear hyperkeratosis of the volar aspects of the fingers and sometimes the palms. SPPK is due to defects in desmosomes, the major epithelial intercellular adhesion junctions, which confer strength and rigidity to tissues that experience high mechanical stress. The disease displays locus heterogeneity with causative mutations in four genes – the desmosomal proteins desmoglein 1 (*DSG1*) and desmoplakin (*DSP*), keratin 1 (*KRT1*) and keratin 16 (*KRT16*) having been reported.^3^, ^4^, ^5^, ^6^

We report one American, one African and five European unrelated families with autosomal dominant PPK, owing to one recurrent and five novel *DSG1* mutations. These demonstrate phenotypic variation despite a common molecular basis.

## Case reports

Families 1, 2 and 7 presented to their local dermatology services. Families 3–6 were identified through the International Pachyonychia Congenita Research Registry. All reported having PPK since early childhood, with a positive family history in pedigrees 1–6. The proband in family 7 was the only known affected family member. No affected individuals had any history of skin fragility, blistering, hair or cardiac abnormalities.

**Figure 1:**
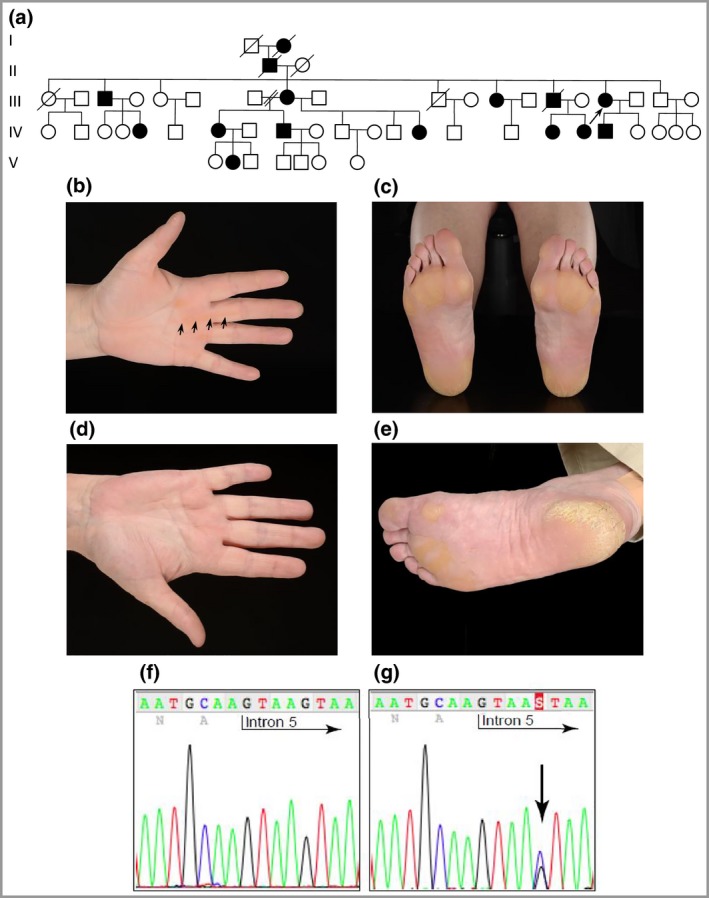
Pedigree, clinical images and mutation analysis of family 1 (Scottish). (a) Pedigree showing a history of palmoplantar keratoderma. The arrow indicates the proband. (b) Striate hyperkeratosis (arrows) of the proband's digits and palm. (c) Soles of the proband showing focal hyperkeratosis. (d) Normal palm of the proband's sister. (e) Soles of the subject in (d) showing fissuring plantar hyperkeratosis. (f) DNA sequence of the desmoglein 1 gene (*DSG1*) in an unaffected control sample. (g) The same region of *DSG1* from the proband. The arrow indicates the novel splice‐site mutation between exon 5 and intron 5: c.517+5G>C, resulting in a frameshift and premature stop codon, p.Tyr126Hisfs*2.

**Figure 2:**
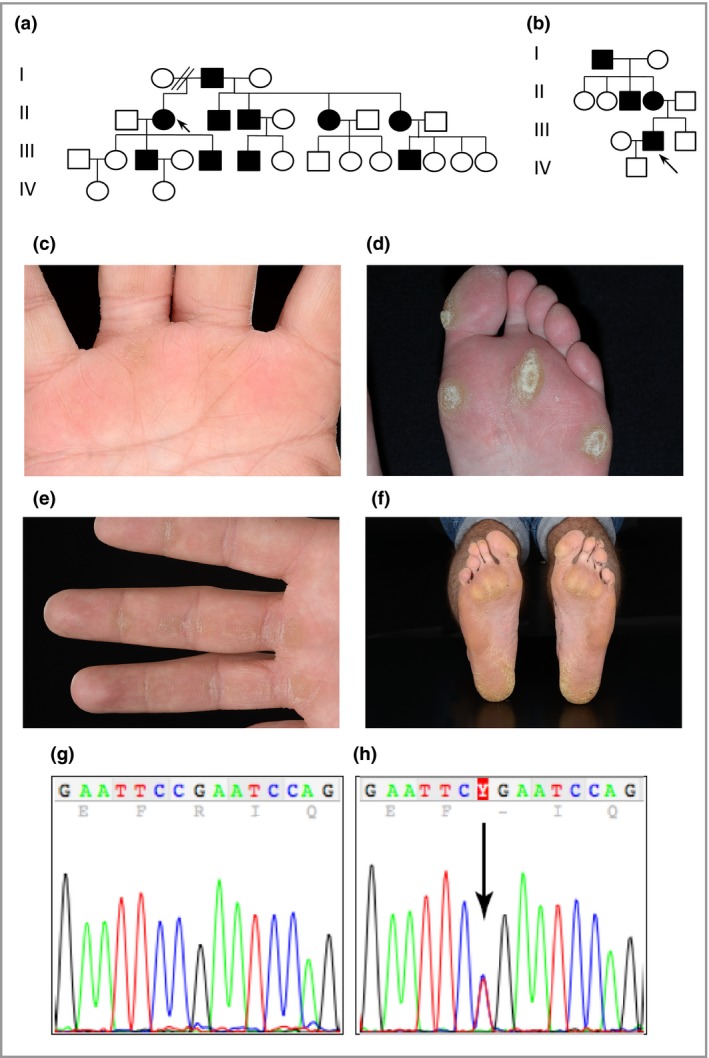
Pedigrees of families 2 (Scottish) and 3 (English), clinical images of family 2 and mutation analysis of families 2 and 3. (a) Pedigree of family 2 showing a history of palmoplantar keratoderma. The arrow indicates the proband. (b) Pedigree of family 3 showing a history of palmoplantar keratoderma. (c) Palm of the proband's half‐sister in family 2 showing focal hyperkeratosis (arrows). (d) Focal plantar hyperkeratosis of the proband's forefoot in family 2. (e) Fingers of the proband's son in family 2 showing linear hyperkeratosis (arrows) on the volar surface of the digits with focal palmar thickening. (f) Soles of the subject in (e) showing focal plantar hyperkeratosis. (g) DNA sequence of exon 2 of the desmoglein 1 gene (*DSG1*) in an unaffected control sample. (h) The same region of *DSG1* from the probands of family 2 and 3. The arrow indicates a heterozygous C>T mutation at c.76 resulting in a premature termination codon at p.Arg26*.

**Figure 3:**
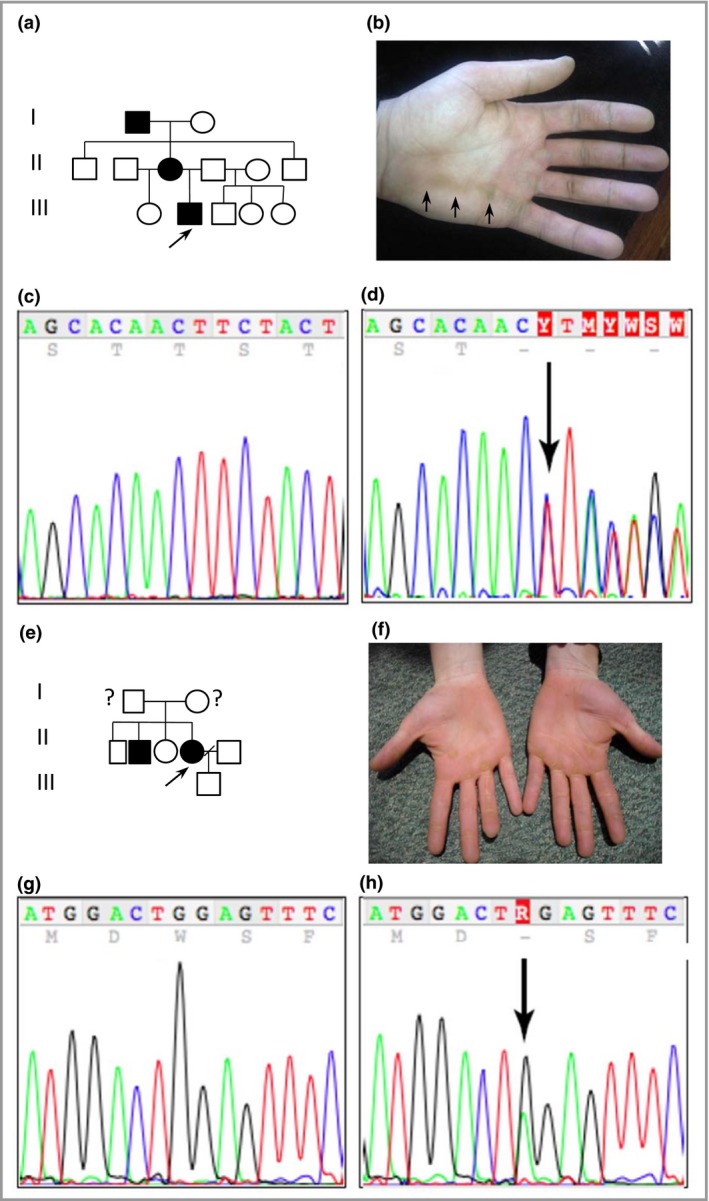
Pedigree, clinical images and mutation analysis of families 4 (Norwegian) and 5 (Northern Irish). (a) Pedigree of family 4 showing a history of palmoplantar keratoderma. The arrow indicates the proband. (b) Striate palmar hyperkeratosis (arrows) of the proband in family 4. (c) DNA sequence of exon 11 of the desmoglein 1 gene (*DSG1*) in an unaffected control sample. (d) The same region of *DSG1* from the proband in family 4; the arrow indicates a novel mutation c.1560‐1561del resulting in a frame shift at Ser521Tyrf*2. (e) Pedigree of family 5. The arrow indicates the proband; no clinical information was available regarding her parents. (f) Striate pattern on the digits with focal palmar hyperkeratosis of the proband in family 5, who had a focal pattern on the soles of the feet. (g) DNA sequence of exon 1 of *DSG1* in an unaffected control sample. (h) The same region of *DSG1* from the proband of family 5. The arrow indicates a novel mutation c.8G>A, resulting in a premature termination codon at p.Trp3*.

**Figure 4:**
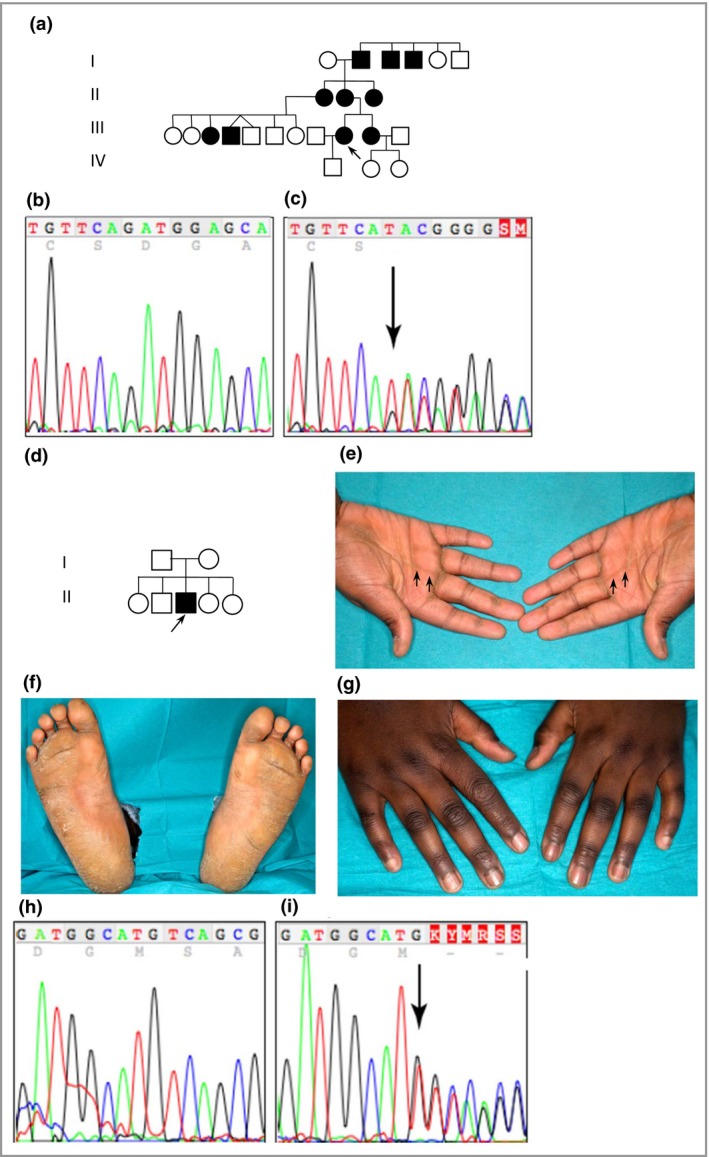
Pedigree and mutation analysis of families 6 (American) and case 7 (Ghanaian). (a) Pedigree of family 6 showing a history of palmoplantar keratoderma. The arrow indicates the proband, who had a striate digital pattern with diffuse keratoderma of the soles. (b) DNA sequence of exon 12 of the desmoglein 1 gene (*DSG1*) in an unaffected control sample. (c) The same region of *DSG1* from the proband of family 6, the arrow indicates a novel mutation c.1771_1784del14, resulting in p.Asp591Phefs*9. (d) Pedigree of case 7. The arrow indicates the proband. (e) Striate palmar hyperkeratosis (arrows) of the proband in family 7. (f) Soles of the proband showing diffuse hyperkeratosis. (g) Knuckle pads over the proband's dorsal interphalangeal joints. (h) DNA sequence of exon 7 of *DSG1* in an unaffected control sample. (i) The same region as in (g) from the proband of family 7. The arrow indicates the heterozygous mutation c.746dupT, resulting in p.Met249Ilefs*6.

In family 1, the proband had striate keratoderma of the digits and palms, with a focal pattern on the soles (Fig. 1a–c). Her sister and niece had no palmar involvement, but exhibited isolated focal keratoderma of the soles (Fig. 1d, e). In family 2, the proband and her half‐sister had focal palmar and plantar hyperkeratosis (Fig. 2a, c, d). The proband's 24‐year‐old son, a soldier, had striate hyperkeratosis of the digits, with focal palmar and plantar involvement (Fig. 2e, f). Mild hyperkeratosis of the elbows and knees was present in all three individuals. Families 3–7 had striate patterns on the palms and/or digits, with focal or diffuse plantar keratoderma (Figs. 2b, 3, 4). Furthermore, the probands in families 3 and 7 had mild hyperkeratosis over the dorsal interphalangeal joints, and knuckle (Fig. 4g) and toe pads, respectively. A biopsy of nonpalmoplantar skin for histological assessment was declined by all patients. Two individuals reported mild plantar pain when the keratoderma was very thick and unpared, but this was not a significant feature in the other cases. See Table S1 (see Supporting Information) for a clinical summary.

Blood or saliva was obtained, following written informed consent and ethical approval by a Western Institutional Review Board that complies with the Declaration of Helsinki (File S1; see Supporting Information). Polymerase chain reaction amplification was performed and Sanger sequencing was employed to screen the exons and exon/intron boundaries of *DSG1*, using primers as previously described.^3^

Family 1 was screened directly for *DSG1* mutations, as the proband presented with SPPK. Families 2–6 were initially screened for pachyonychia congenita mutations in keratin genes *KRT6A*,* KRT6B*,* KRT6C*,* KRT16* and *KRT17*. In family 7, screening for *KRT1* and *KRT9*, associated with diffuse PPK, was negative; whole‐exome sequencing of the proband and his unaffected parents was performed (File S1; see Supporting Information). In all cases, nonpathogenic variants were excluded through sequencing unaffected family members, and by reference to the Database of Single Nucleotide Polymorphisms, the 1000 Genome Project and the Exome Variant Server.

Affected members in family 1 had a novel splice‐site mutation in *DSG1* between exon 5 and intron 5 (c.517+5G>C). Total RNA was extracted from a skin biopsy, and a cDNA fragment spanning exons 4–7 was amplified (File S1; see Supporting Information). DNA sequencing revealed very low levels of expression of the mutant allele, suggesting significant nonsense‐mediated mRNA decay. This mutation leads to skipping of exon 5, resulting in a frameshift and premature stop codon, p.Tyr126Hisfs*2 (Fig. 1f, g). Families 2 and 3 were heterozygous for a previously described nonsense mutation in *DSG1* in exon 2 (c.76C>T, p.Arg26*) (Fig. 2g, h).^3^ Affected members in family 4 had a novel *DSG1* mutation c.1560‐1561del in exon 11, resulting in p.Ser521Tyrfs*2 (Fig. 3c, d). A novel heterozygous nonsense mutation*,* c.8G>A resulting in p.Trp3* in exon 1 (Fig. 3g, h) was found in family 5. Family 6 had a novel mutation, c.1771_1784del14. This 14 base pair deletion results in p.Asp591Phefs*9 in exon 12 (Fig. 4b, c). Lastly, the proband in family 7 had a novel *DSG1* duplication mutation, c.746dupT, which results in a frameshift and premature stop codon, p.Met249Ilefs*6 (Fig. 4h, i).

## Discussion

There are three types of SPPK – type 1 (OMIM 148700) is the most common, caused by mutations in *DSG1*. SPPK types 2 (OMIM 612908) and 3 (OMIM 607654) are caused by desmoplakin and keratin‐1 mutations, respectively. Desmoplakin mutations can be associated with cardiac disease and woolly hair, but extracutaneous manifestations are not described with *DSG1* mutations. Pachyonychia congenita rarely presents with striate PPK; Almutawa *et al*. reported a recurrent mutation in *KRT16*, causing striate palmar and diffuse plantar keratoderma, nail thickening and knuckle pads.^6^ Mutations in other *DSG1* binding partners, resulting in varying PPK phenotypes, have also been described.^1^ To date, 23 mutations in *DSG1* have been reported in 25 families with PPK; the majority had striate palmar and focal plantar keratoderma, but focal^7^ or diffuse^8^ palmar patterns have also been described. A more severe phenotype, severe dermatitis, multiple allergies, and metabolic wasting (SAM) syndrome, has been described in individuals with biallelic *DSG1* mutations^9^ and in one case with a heterozygous mutation in desmoplakin.^10^

We report the largest series of *DSG1* mutations to date; two nonsense mutations (c.76C>T; p.Arg26* and c.8G>A; p.Trp3*), one duplication (c.746dupT; p.Met249Ilefs*6), two deletions (c.1560_1561del; p.Ser521Tyrfs*2 and c.1771_1784del14; p.Asp591Phefs*9), and one splice‐site mutation (c.517+5G>C; p.Tyr126Hisfs*2). All were autosomal dominant heterozygous mutations.

Reported *DSG1* mutations causing SPPK are thought to result in haploinsufficiency through nonsense‐mediated mRNA decay. In SAM syndrome, there is a near‐total absence of *DSG1* from the cell membrane,^9^, ^10^, ^11^ as a result of either nonsense‐mediated mRNA decay,^11^ failure of *DSG1* localization^9^ or decreased DSG1 expression.^10^ It therefore seems reasonable to hypothesize that any reported SPPK *DSG1* mutation might, if biallelic, result in SAM syndrome. Harmon *et al*. demonstrated that *DSG1* interacts with Erbin, downregulating the Ras/MAPK pathway. Elevated Ras activity resulting from absent or insufficient *DSG1* is postulated as a mechanism underlying SPPK. The high rate of PPK in disorders of the Ras/MAPK pathway supports this.^12^

*DSG1* PPK phenotypes are restricted to areas of high pressure and abrasion.^3^ Keratoderma can be exaggerated by environmental factors, which may explain the striate pattern in the soldier in family 2, when other family members had isolated focal PPK. The mechanism for this is unclear, but may be a result of reduced desmosome function,^3^, ^5^ variations in desmosome configuration and protein expression,^13^ or a relative lack of coping with/adapting to environmental stress in desmoglein‐1 haploinsufficient individuals. Nonpalmoplantar hyperkeratotic plaques, as described in families 2 and 3, have been reported in three other families with *DSG1* mutations.^7^, ^14^ The proband in family 7 is the first reported case to have knuckle and toe pads.

Our cases demonstrate phenotypic heterogeneity despite their unifying molecular basis. Intrafamilial PPK variation has been reported with *DSG1* mutations,^8^, ^15^, ^16^ but the extent may be underestimated. In family 1, only the proband had striate palmar PPK; the two other affected relatives had no palmar disease. In family 2, the proband had focal palmoplantar disease, and it was only when another relative presented with striate palmar disease that *DSG1* screening was performed, which highlights the importance of examining multiple family members. The recurrent mutation in families 2 and 3 (c.76C>T) has been reported in three other pedigrees – sporadic striate PPK,^3^ diffuse nonepidermolytic PPK,^8^ and striate palmar and focal plantar keratoderma.^17^ *DSG1* screening has hitherto been performed primarily for striate PPK. Given the variety of phenotypes, and the difficulty in distinguishing these clinically, we suggest a low threshold for *DSG1* screening in PPK where initial keratin gene screening has been negative.

## Supporting information


**Table S1.** Summary of desmoglein 1 gene (*DSG1*) mutations and clinical features.

**File S1.** Supplementary methods.
