# Supplementary material for: Mutations in desmoglein 1 cause diverse inherited palmoplantar keratoderma phenotypes: implications for genetic screening
Source: Br J Dermatol. 2017 Apr 2;176(5):1345–50. doi: 10.1111/bjd.14973 (PMC5485079; doi:10.1111/bjd.14973)
Supplement: Supplementary file 1 — Table S1. Summary of desmoglein 1 gene (DSG1) mutations and clinical features. [file BJD-176-1345-s001.doc]

**Supplementary Material**

**Table S1: Case Reports**

**File S1: Methods**

Genomic DNA was extracted from peripheral blood leucocytes using standard procedures or from saliva collected in an Oragene DNA sample collection kit (DNA Genotek, Ontario, Canada) and extracted according to the manufacturer’s protocol.

*Whole exome sequencing*

The extracted DNA for the samples was quality controlled for quantity and purity with a Qubit Fluorometer (Thermo) and libraries were prepared via the NimbleGen SeqCap v3 whole exome sequencing protocol as per the manufacturer's instructions by the Genomic Sequencing Facility (Dundee). The libraries were sequenced on an Illumina HiSeq2000 instrument using a 100bp paired-end strategy generating between 86-110 million reads which were checked with FastQC and FastQ Screen.

The exome data were processed as per the GATK best practices manual. Briefly, reads were aligned against the GRCh37 human genome with bowtie (v2.1.0), PCR duplicates removed with Picard tools MarkDuplicates (v1.89) and realignment and recalibration with GATK v2.2-8-g99996f2. Variants were called with GATK's UnifiedGenotyper and annotated with Ensembl's Variant Effect Predictor (v72) [1, 2]. Annotated variants were stored in a MySQL database and specific queries made to take advantage of known hereditary patterns in the family and to filter out intractable variants such as synonymous and non-coding region variants.

*RNA extraction, reverse transcription, PCR and sequencing*

RNA was extracted from a skin biopsy using the Qiagen RNeasy Plus mini kit (Qiagen, Crawley, UK). The skin biopsy was disrupted using the Qiagen TissueLyser II.  RNA was reverse transcribed into cDNA using the Applied Biosystems High Capacity cDNA reverse transcription kit.
PCR reactions were set up in Qiagen Coral Load PCR buffer containing 1.5mM MgCl_2_ and 1U HotStarTaq *Plus* DNA Polymerase (Qiagen, Crawley, UK) with forward 5' GGAGTAGGAATTGATCAGCCA 3' and reverse 5' AGAGCCTCTTACAGCAAGAGC 3' primers. The following PCR conditions were used (95°C 5 min) x1; (94°C 1 min, 60°C 1 min, 72°C 1 min) x 35; and (72°C 10 min) x 1. PCR products were purified using QiaQuick PCR spin columns (Qiagen House, Crawley, UK) and sequenced on an ABI 3730 Automated DNA sequencing machine (Foster City, CA) according to the manufacturer’s instructions.

**Supplement References**

1. McKenna A, Hanna M, Banks E *et al.* The Genome Analysis Toolkit: a MapReduce framework for analyzing next-generation DNA sequencing data. *Genome Research* 2010; **20**: 1297-303.

2. McLaren W, Pritchard B, Rios D *et al.* Deriving the consequences of genomic variants with the Ensembl API and SNP Effect Predictor. *Bioinformatics* 2010; **26**: 2069-70.
