# Supplementary material for: Mutations in desmoglein 1 cause diverse inherited palmoplantar keratoderma phenotypes: implications for genetic screening
Source: Br J Dermatol. 2017 Apr 2;176(5):1345–50. doi: 10.1111/bjd.14973 (PMC5485079; doi:10.1111/bjd.14973)
Supplement: Supplementary file 2 — File S1. Supplementary methods. [file BJD-176-1345-s002.ppt]

## Slide 1
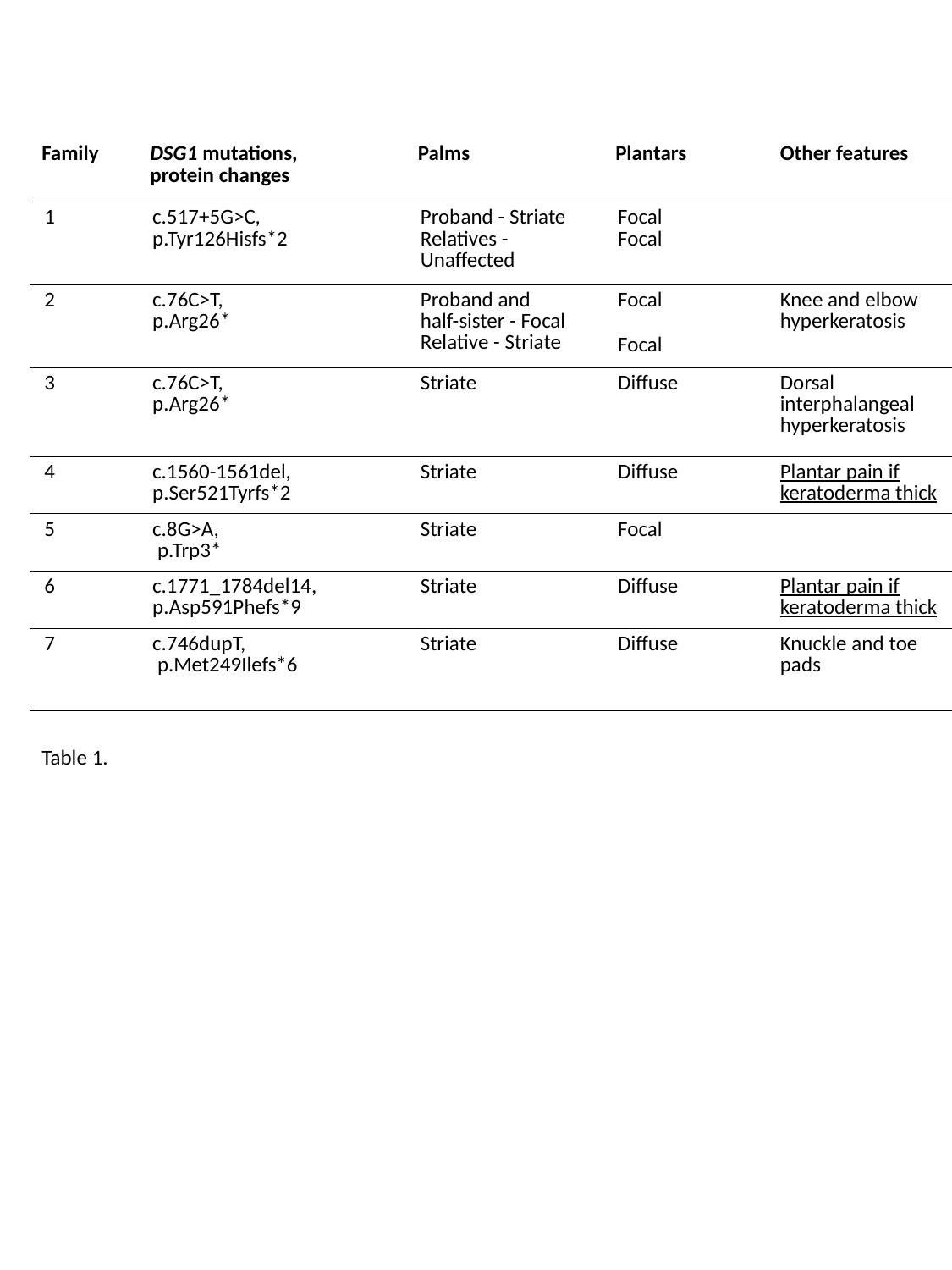

| Family | DSG1 mutations, protein changes | Palms | Plantars | Other features |
| --- | --- | --- | --- | --- |
| 1 | c.517+5G>C, p.Tyr126Hisfs\*2 | Proband - Striate Relatives - Unaffected | Focal Focal | |
| 2 | c.76C>T, p.Arg26\* | Proband and half-sister - Focal Relative - Striate | Focal Focal | Knee and elbow hyperkeratosis |
| 3 | c.76C>T, p.Arg26\* | Striate | Diffuse | Dorsal interphalangeal hyperkeratosis |
| 4 | c.1560-1561del, p.Ser521Tyrfs\*2 | Striate | Diffuse | Plantar pain if keratoderma thick |
| 5 | c.8G>A, p.Trp3\* | Striate | Focal | |
| 6 | c.1771\_1784del14, p.Asp591Phefs\*9 | Striate | Diffuse | Plantar pain if keratoderma thick |
| 7 | c.746dupT, p.Met249Ilefs\*6 | Striate | Diffuse | Knuckle and toe pads |
Table 1.
